# Quality of life in adults treated in infancy for hydrocephalus

**DOI:** 10.1007/s00381-014-2425-4

**Published:** 2014-04-23

**Authors:** Barbro Lindquist, Elisabeth Fernell, Eva-Karin Persson, Paul Uvebrant

**Affiliations:** 1 Department of Habilitation, Halmstad County Hospital, Halmstad, Sweden; 2 Unit of Neurodevelopmental Disorders, Skaraborg Hospital Mariestad, Mariestad, Sweden; 3 Gillberg Neuropsychiatry Center, Institute of Neuroscience and Physiology, University of Gothenburg, Gothenburg, Sweden; 4 The Queen Silvia Children’s Hospital/Sahlgrenska University Hospital, Institute of Clinical Sciences, University of Gothenburg, SE-416 85 Gothenburg, Sweden

**Keywords:** Infantile hydrocephalus, Quality of life, Prognosis, Long-term follow-up

## Abstract

**Purpose:**

The objective was to analyze quality of life in a very long-term follow-up study of now adult individuals, treated for hydrocephalus (without spina bifida) during infancy.

**Methods:**

The entire series was population-based, and the subgroup under study consisted of the 29 individuals without intellectual disability, who consented to participate. About one third had concomitant mild cerebral palsy or epilepsy or both. A Finnish validated questionnaire, the 15D, was used to measure quality of life.

**Results:**

There was no significant difference between the study group and the controls with regard to the total quality of life score. Individuals with associated cerebral palsy and/or epilepsy had a lower total score compared with both those without associated impairments and controls. Most participants differed from controls in the dimension of mental/memory function which pertains to executive functions, an ability of considerable importance for daily life skills.

**Conclusion:**

It is important to follow children with hydrocephalus over time—due to the different etiological panorama, interventions, and associated impairments this group displays. This is the only way to learn more about critical factors that require attention and that predict quality of life in adulthood.

## Introduction

Various aspects of hydrocephalus have been studied during the past 30–40 years, i.e., since the introduction of modern shunt treatment that has profoundly reduced mortality. Epidemiological, clinical, neuropsychological, and neurosurgical follow-up studies have been presented [2, 7, 12, 14, 16, 21, 22]. The latest step in this development has been an emerging interest in the health-related quality of life of these individuals [8–10].

In children with hydrocephalus, the increased intracranial pressure, regardless of etiology, leaves them at risk of developing cognitive disabilities, epilepsy, and cerebral palsy [11, 16]. In a population-based study of children with infantile hydrocephalus, we previously reported that 42 % had no signs of neurological deficits, while about one third had cerebral palsy, one third epilepsy, and 40 % mental retardation/intellectual disability (IQ <70) [11, 16]. Even those with a normal or slightly subnormal IQ often had significant difficulties in learning, memory and other executive functions [12].

The term “quality of life” generally refers to the subjective evaluation of well-being in individuals in terms of health and social factors. The concept may be viewed from various perspectives; social, economic, work related, and related to health. In health-related measurements, there are differences in the subjectively perceived quality of life depending on whether an illness is congenital, acquired at an early stage or acquired later in life. Surviving a trauma may generate an experience of high quality of life under circumstances that would hardly be acceptable to another person. It is therefore important that measurements of quality of life are based on self-reports. Kulkarni et al. [8] developed a measurement of quality of life, the HOQ—Hydrocephalus Outcome Questionnaire. In a study of children with hydrocephalus [9] using the HOQ, it was shown that shunt infections and other shunt-related complications, as well as a high frequency of epileptic seizures and a longer distance from home to hospital, were some of the predictors of poor quality of life as estimated by parents. In a recent review, Kulkarni [10] also concluded that quality of life questions should be a standard part of clinical and research practice.

There are few studies dealing with the long-term effect of early-onset hydrocephalus on the quality of life in adults. Gupta et al. [4] examined social and functional outcomes at the age of 20 years or more in patients treated for early-onset hydrocephalus and found that depression, requiring treatment, had occurred in two thirds of those with a diagnosis of hydrocephalus before 18 months of age and that social functioning was more impaired in individuals with this early onset. In another follow-up study, individuals aged below 15 years, treated for hydrocephalus of various etiologies and with different clinical presentations, answered a questionnaire about quality of life, the SF-36 [15]. In this group, quality of life was found to be slightly lower than in the normative population. A significant difference between individuals with hydrocephalus and the reference group was found with regard to physical functioning and general health.

The aim of this study was to investigate the self-reported quality of life in a population of adults who had been treated for hydrocephalus during their first year of life and who had been assessed as not mentally retarded when they were children.

## Methods

### Study group

From a series of children with infantile hydrocephalus, born in the western-Swedish health-care region, those born in 1967–1978 participated in a clinical follow-up study in the early 1980s [3]. The total group comprised 68 children, 61 of whom had been treated with a shunt. Forty-three of these 61 children had had a normal IQ (IQ above 70) according to cognitive testing at school age and should therefore be able reliably to evaluate their life situation when answering a questionnaire as adults. These 43 individuals were invited to take part in a follow-up study of medical conditions and social and employment status [17], cognitive functioning [13], and the present study, focusing on quality of life. Twenty-nine (18 men and 11 women) of the 43 individuals (67 %) agreed to participate. The mean age of the study group was 34 years (range 30–41), and the median IQ was 101 (range 83–120) [13]. Five participants had a diagnosis of epilepsy, three with a mild form of cerebral palsy, and one had a combination of epilepsy and mild cerebral palsy [17].

### Instrument

A well-documented health-related quality of life instrument, the 15D [19], was selected for this study. It is self-administered and can be used both as a profile and as a single index score measurement. The 15D questionnaire consists of 15 dimensions: mobility (move), vision (see), hearing (hear), breathing (breath), sleeping (sleep), eating (eat), speech (speech), elimination (elim), usual activities (uact), mental function (mental), discomfort and symptoms (disco), depression (depr), distress (distr), vitality (vital), and sexual activity (sex). Each dimension is divided into five levels, by which more or less of the attribute can be distinguished; from 1 (corresponds to normal function) to 5 (corresponds to total dependency). For each presented dimension, the individual scores the level that best accords with his/her health status. The dimension “usual activities” refers to work, studies, work in the household, and leisure time activities. “Mental function” contains questions about memory function and the ability to think clearly and consistently and “Discomfort and symptoms” includes questions about pain, nausea, and similar complaints.

The 15D scores as reported by the study group were mailed to Professor H Sintonen, University of Helsinki, Department of Public Health, for statistical evaluation and comparison with a group of 1,613 controls matched for age and gender.

The maximum score (normal) is 1.0. A difference of 0.03 in the 15D score has been found to be clinically important in the sense that people on average can experience the difference.

### Statistics

The Mann-Whitney test was used to analyze the results.

### Ethics

The study was approved by the Research Ethics Committee at Gothenburg University.

## Results

**Fig. 1:**
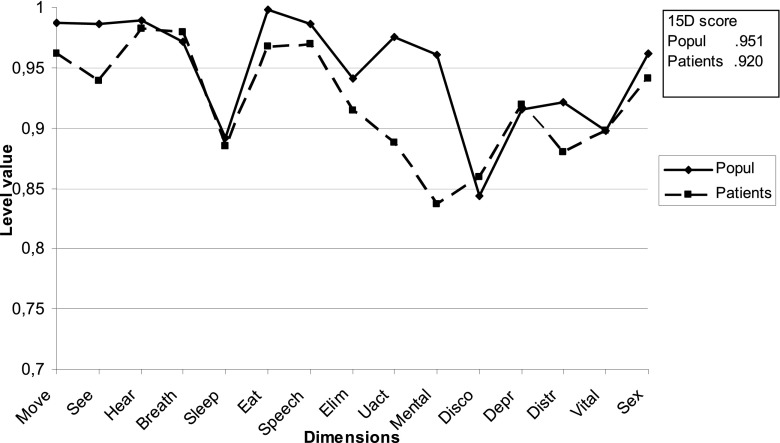
Health-related quality-of-life profiles in 29 individuals with hydrocephalus and 1,613 controls. Mobility (*move*), vision (*see*), hearing (*hear*), breathing (*breath*), sleeping (*sleep*), eating (*eat*), speech (*speech*), elimination (*elim*), usual activities (*uact*), mental function (*mental*), discomfort and symptoms (*disco*), depression (*depr*), distress (*distr*), vitality (*vital*), and sexual activity (*sex*)

**Fig. 2:**
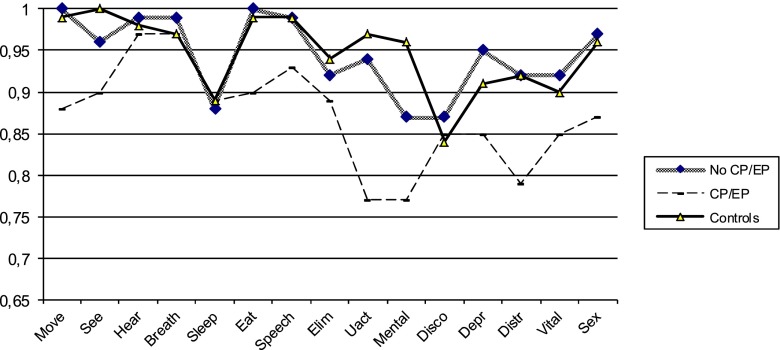
Health-related quality-of-life profiles in nine individuals with cerebral palsy (*CP*) and/or epilepsy (*EP*), 20 without cerebral palsy or epilepsy, and 1,613 controls. Mobility (move), vision (*see*), hearing (*hear*), breathing (*breath*), sleeping (*sleep*), eating (*eat*), speech (*speech*), elimination (*elim*), usual activities (*uact*), mental function (*mental*), discomfort and symptoms (*disco*), depression (*depr*), distress (*distr*), vitality (*vital*), and sexual activity (*sex*)

The average 15D score of the individuals who had been treated with a shunt due to infantile hydrocephalus was 0.92 (*n* = 29), while that of the controls was 0.95 (*n* = 1,613) (n.s.). The group with hydrocephalus had significantly lower results than controls in dimensions pertaining to vision (*p* = 0.001), eating (*p* = 0.000), usual activities (*p* = 0.004), and mental function (*p* = 0.000), (Fig. 1, Table 1). Most of the differences were due to the subgroup of nine individuals with additional signs of brain impairment, i.e., with cerebral palsy and/or epilepsy. The average score in this group of nine individuals was 0.87 compared with 0.94 in the group without such associated impairments. Individuals with cerebral palsy and/or epilepsy differed most in dimensions relating to mobility, vision, eating, usual activities, distress, and vitality (Fig. 2, Table 2). One individual with major visual problems obtained low scores on all these dimensions. Two of four individuals with cerebral palsy and four of five with epilepsy reported memory problems. Of the five individuals who described problems with usual activities, three had cerebral palsy. Of the six who reported problems with vision, one had cerebral palsy, one had epilepsy, and both with eating difficulties had cerebral palsy.

**Table 1:** Health-related quality of life scores in 29 individuals with hydrocephalus (HC) and 1,613 controls

Dimension	HC mean (SD)	Control mean (SD)	*p* value
Mobility	0.96 (0.12)	0.99 (0.07)	0.055
Vision	0.94 (0.15)	1.0 (0.06)	0.001
Hearing	0.98 (0.07)	0.98 (0.06)	0.431
Breathing	0.98 (0.08)	0.97 (0.01)	0.751
Sleeping	0.88 (0.19)	0.89 (0.15)	0.792
Eating	0.97 (0.92)	0.99 (0.68)	0.000
Speech	0.97 (0.92)	0.99 (0.68)	0.141
Elimination	0.91 (0.18)	0.94 (0.14)	0.559
Usual activities	0.89 (0.24)	0.97 (0.10)	0.004
Mental function	0.84 (0.23)	0.96 (0.12)	0.000
Discomfort and symptoms	0.86 (0.22)	0.84 (0.17)	0.235
Depression	0.92 (0.16)	0.91 (0.14)	0.720
Distress	0.88 (0.18)	0.92 (0.15)	0.196
Vitality	0.90 (0.20)	0.90 (0.15)	0.518
Sexual activity	o.94 (0.14)	0.96 (0.12)	0.304

**Table 2:** Health-related quality of life in nine individuals with hydrocephalus and cerebral palsy (CP) and/or epilepsy (EP), 20 without cerebral palsy or epilepsy, and 1,613 controls

Dimension	CP/EP mean	No CP/EP mean	Control mean
Mobility	0.88	1.00	0.99
Vision	0.90	0.96	1.0
Hearing	0.97	0.99	0.98
Breathing	0.97	0.99	0.97
Sleeping	0.89	0.88	0.89
Eating	0.90	1.00	0.99
Speech	0.93	0.99	0.99
Elimination	0.89	0.92	0.94
Usual activities	0.77	0.94	0.97
Mental function	0.77	0.87	0.96
Discomfort and symptoms	0.85	0.87	0.84
Depression	0.85	0.95	0.91
Distress	0.79	0.92	0.92
Vitality	0.85	0.92	0.90
Sexual activity	0.87	0.97	0.96

The subgroup of 20 participants without neuroimpairments (cerebral palsy and/or epilepsy) did not differ significantly from the controls; their 15D score was 0.94 vs. 0.95. However, they had more problems than controls in terms of mental function, i.e., memory; 15D score 0.87 vs. 0.96, usual activities score 0.94 vs. 0.97, and vision score 0.96 vs. 1.0.

## Discussion

This study of quality of life is part of a very long-term follow-up of a population-based group, treated in infancy for hydrocephalus without concomitant spina bifida [13, 17]. The study group included the 29 individuals who had been found to have an IQ within the normal distribution and who consented to participate in the follow-up study. However, nine of them had mild cerebral palsy, epilepsy, or both impairments. One limitation of this study is that we have no detailed information about the 14 individuals that declined participation. However, they did not differ with respect to age, gender, and the etiology of their hydrocephalus [13].

This study of quality of life, as measured with the 15D questionnaire, revealed that this group of normally gifted adults rated themselves as having quality of life as good as that of controls, with the exception of those with additional neurological impairments in the form of cerebral palsy and epilepsy. Despite this overall good quality of life, even those without additional motor neuroimpairments or epilepsy still had problems in the dimensions of memory, usual activities, and vision.

The 15D instrument, developed in Finland, has been widely used in several countries, and rigorous comparisons based on eleven evaluation studies from six countries have revealed that there is a considerable degree of agreement between health status evaluations [20]. In a previous Swedish study of quality of life 10 years after traumatic brain injury, nine of the 15 dimensions in the 15D deviated significantly in the traumatic brain injury group compared with controls [6]. In our study group, the average 15D score was 0.93 in the total group, 0.87 in the group with cerebral palsy or epilepsy, and 0.94 in individuals without neurological disabilities, indicating that additional brain injury has an impact on coping with vital functions that are important for health-related quality of life.

In the present study, most participants differed from controls in the dimension of mental function, which mainly includes questions pertaining to memory function. Memory deficits have previously been reported in studies of both children and adults with hydrocephalus [5, 12, 18, 24]. This finding was in accordance with the results of the previous assessment of the participants using the WAIS-III, revealing deficits in executive abilities [13, 23]. This function is important in daily life in order to maintain control and structure in the household, at work and in social networks. It was therefore plausible that the dimension of “usual activities,” containing questions about work, studies, household, and spare time, also produced lower results than that of controls. In spite of this, one third had attended university compared with 41 % in the population and two thirds worked full time [17]. In the Norwegian study [15] of young adults with shunt-treated hydrocephalus, the 20-year outcomes, including health-related quality of life (HRQOL), were studied. Individuals with myelomeningocele, as well as with learning disabilities, were included. In this group, 56 % were employed in open-market jobs or were still students, 23 % had sheltered employment, and 21 % were unemployed. The authors reported that the HRQOL was slightly lower in the hydrocephalic cohort compared with the normative population.

In a study from 2012 in which 456 patients were followed, 33.7 % were employed in the competitive labor market. Here, too, patients with different etiological backgrounds and intellectual function were included [21].

The finding of problems in the dimension of vision agreed with the report by Andersson et al. [1] demonstrating very frequent ophthalmological abnormalities in children with hydrocephalus. In the study group, nine of the 29 individuals had neurological impairments, i.e., cerebral palsy, epilepsy, or both. Compared with the 20 without these additional problems, this subgroup had more difficulties in the dimensions of mobility, vision, eating, usual activities, distraction, and vitality—again indicating the major impact of neurological impairments that may accompany hydrocephalus.

The participants in the present study group did not generally describe problems with depression. This was only present in the subgroup with cerebral palsy or epilepsy. In the study by Gupta et al. [4], also including participants with varying cognitive abilities and additional impairments, depression requiring treatment had occurred in as many as 45 %. While our study was based on self-reports from individuals who all had an IQ in the normal range, information on depression in the study by Gupta et al. was supplied either by the patients or by their guardians.

Measuring quality of life with another instrument, the SF-36, revealed poorer perceived health in the domains of physical functioning and general health in young adults with hydrocephalus treated at an early stage [15]. In this group, 18 % had spina bifida, which automatically leads to problems in these areas. The SF-36 does not include questions on memory functions, which are of central interest in patients with hydrocephalus. However, the clustered mental health questions (vitality, mental health, role problems due to emotional problems, and social functioning) did not differ significantly from those of the population in general. In the study by Paulsen [15], as well as in this present study, it was concluded that a poorer outcome in quality of life was related to the presence of additional disorders/injuries to the brain.

This study revealed a reported quality of life that was better than that in the few earlier studies of adults with hydrocephalus [4, 7, 15]. This is probably related to the fact that our study group did not include children with intellectual disability and another possible explanation could be that our study included adults treated as early as in the late 1960s/early 1970s. At that time, few children born extremely preterm survived and our study group only included one child born extremely preterm [17]. Later, in the 1980s and 1990s, prematurity with the ensuing risk of hemorrhage and thereby an increased risk of more extensive brain lesions had become a more frequent etiological background [16]. If the proportion of participants with more severe brain injuries had been larger in the present follow-up, the results may not have been so favorable.

It is encouraging that our study group of adults with shunt-treated hydrocephalus perceived that they had a good quality of life and that the majority were able to cope with their difficulties related to executive dysfunctions. This was also reflected by their high degree of participation in society, where the majority had an education and employment, as well as partners and children, to a degree that did not differ from adults in general. It is, however, important prospectively to follow children with hydrocephalus—due to different etiologies and with different associated impairments—in order to learn more about the critical factors that require attention and predict their quality of life as adults. The review by Vinchon et al. in 2012 also points out that more prospective and retrospective studies are needed to evaluate the long-term consequences of the treatment of hydrocephalus, including patients with different etiological backgrounds [22].
